# A multicenter, randomized trial comparing efficacy and safety of paclitaxel/capecitabine and cisplatin/capecitabine in advanced gastric cancer

**DOI:** 10.1007/s10120-018-0809-y

**Published:** 2018-02-27

**Authors:** Zhihao Lu, Xiaotian Zhang, Wei Liu, Tianshu Liu, Bing Hu, Wei Li, Qingxia Fan, Jianming Xu, Nong Xu, Yuxian Bai, Yueyin Pan, Qing Xu, Wei Bai, Li Xia, Yong Gao, Wenling Wang, Yongqian Shu, Lin Shen

**Affiliations:** 1 Key Laboratory of Carcinogenesis and Translational Research (Ministry of Education/Beijing), Department of Gastrointestinal Oncology, Peking University Cancer Hospital and Institute, #52 Fucheng Road, Haidian District, Beijing, 100142 People’s Republic of China; 2 Department of Medical Oncology, The 4th Hospital, Hebei Medical University, Shijiazhuang, Hebei China; 3 Department of Oncology, Shanghai Zhong Shan Hospital, Fu Dan University, Shanghai, China; 4 Department of Oncology, Anhui Provincial Hospital, Hefei, Anhui China; 5 Department of Oncology, The First Hospital of JiLin University, Changchun, Jilin China; 6 Department of Oncology, The First Affiliated Hospital of Zhengzhou University, Zhengzhou, Henan China; 7 Department of Gastrointestinal Oncology, Affiliated Hospital Cancer Center, Academy of Military Medical Sciences, Beijing, Dongda Street 8, Fengtai District, Beijing, 100071 People’s Republic of China; 8 The First Affiliated Hospital of Zhejiang University School of Medicine, Hangzhou, Zhejiang China; 9 Gastrointestinal Oncology Department, Harbin Medical University Cancer Hospital, Harbin, Heilongjiang China; 10 Department of Medical Oncology, The First Affiliated Hospital of Anhui Medical University, Hefei, China; 11 Department of Medical Oncology, Shanghai Tenth People’s Hospital, Tongji University, Shanghai, China; 12 Department of Gastrointestinal Oncology, Shanxi Cancer Hospital, Xinghua District, Taiyuan, Shanxi China; 13 Department of Medical Oncology, Jilin Province People’s Hospital, Changchun, Jilin China; 14 Department of Medical Oncology, Shanghai Dongfang Hospital, Tongji University, Pudong New District, Shanghai, China; 15 Department of Abdominal Oncology, Guizhou Cancer Hospital, Yunyan District, Guiyang, Guizhou China; 16 Department of Medical Oncology, Jiangsu Province Hospital, Nanjing, Jiangsu China

**Keywords:** Stomach neoplasms, Paclitaxel, Capecitabine, Cisplatin

## Abstract

**Background:**

We compared efficacy and safety of paclitaxel/capecitabine therapy followed by capecitabine for maintenance (PACX) versus cisplatin/capecitabine therapy (XP) in advanced gastric cancer.

**Methods:**

Multicenter, randomized, phase III trial was conducted in China (December 2009–February 2014). Adults (*n* = 320) with histologically confirmed, untreated metastatic/unresectable gastric or gastroesophageal junction adenocarcinoma; with ≥ 1 measureable lesions according to Response Evaluation Criteria in Solid Tumors 1.0 criteria; Karnofsky performance score ≥ 70 and life expectancy ≥ 3 months were randomized (1:1) to PACX or XP. PACX group received paclitaxel 80 mg/m^2^ intravenous on days 1 and 8; capecitabine 1000 mg/m^2^ orally BD on days 1–14, followed by a 7-day rest interval for 4 cycles, followed by maintenance capecitabine at same dosage/schedule until disease progression, unendurable adverse events or death. XP group received cisplatin intravenous 80 mg/m^2^ on day 1 and capecitabine at same dosage/schedule as PACX group per cycle for 6 cycles.

**Results:**

Median progression-free survival (5.0 versus 5.3 months; hazard ratio [95% CI]: 0.906; 0.706–1.164; *p* = 0.44) and overall survival (12.5 versus 11.8 months; hazard ratio: 0.878 [0.685–1.125]; *p* = 0.30) were not significantly different between PACX and XP groups. Objective response rate was significantly higher (43.1 versus 28.8%; *p* = 0.012) and disease control rate was similar (77.5 versus 72.5%; *p* = 0.75) in PACX versus XP, respectively. Quality of life was significantly improved in PACX versus XP after three treatment cycles. Many treatment-related adverse events were significantly lesser in PACX than XP.

**Conclusions:**

First-line chemotherapy with PACX is effective with milder toxicities in advanced gastric cancer, but could not replace XP.

**Electronic supplementary material:**

The online version of this article (10.1007/s10120-018-0809-y) contains supplementary material, which is available to authorized users.

## Introduction

Gastric cancer is the third most common cancer and the second leading cause of cancer deaths globally. Approximately 50% of the incident cases occur in Eastern Asia (mainly in China) [1]. The median survival of patients with advanced gastric cancer (AGC) is 7–10 months in a majority of large clinical studies [2].

Compared with best supportive care alone, palliative chemotherapy (CT) improves survival and quality of life (QoL) for patients with AGC [2, 3]. In different countries, fluorouracil and cisplatin combination with or without a third drug are most widely used [4]. In the ToGA study, combination therapy of the anti-human epidermal growth factor receptor 2 (HER2) antibody, trastuzumab, with 5-fluorouracil or capecitabine plus cisplatin showed significantly improved survival compared with CT alone in patients with HER2-positive late-stage gastric cancer [5]. However, the toxicities and disadvantages related with use of cisplatin highlight the need for alternative therapeutic options that would maintain good QoL during CT.

Taxanes are considered as alternative first-line chemotherapy options for AGC [4], since they improve survival without compromising QoL. Paclitaxel shows good efficacy and tolerance in AGC either as monotherapy or in combination with other CT drugs [6]. Capecitabine, an oral fluoropyrimidine is rapidly converted to 5-fluorouracil (5-FU) in tumor tissue, and is becoming popular and replacing fluorouracil in AGC for convenience and satisfactory efficacy [7–9]. The randomized phase III non-inferiority trial ML17032 demonstrated that capecitabine can replace 5-FU in AGC combination therapy [10]. Synergistic efficacy mediated by taxane-induced upregulation of thymidine phosphorylase and minimal overlap of major toxicities are advantages of capecitabine and taxane combination [11].

Maintenance therapy with a simplified drug regimen following an intensive CT reduced adverse effects without compromising survival benefit in advanced colorectal cancer [12].

In our previous phase II study, paclitaxel and capecitabine combination as first-line CT with capecitabine maintenance after disease control showed promising efficacy and good tolerance, with median PFS of 188 days and median OS of 354 days, and the median OS in the patient subgroup maintained with capecitabine monotherapy was 531 days [13]. Further randomized trials are required to accurately evaluate the efficacy and safety of this promising regimen for the AGC treatment.

The purpose of this prospective, randomized, controlled phase III study was to compare the efficacy and safety of paclitaxel plus capecitabine regimen as a first-line CT followed by capecitabine monotherapy (PACX) as a maintenance therapy versus cisplatin plus capecitabine (XP) regimen for AGC.

## Methods

### Study design

This was a multicenter, open-label, active-controlled phase III trial conducted in 22 national hospitals with specialized cancer centers across different regions of China from December 2009 to February 2014 (Clinicaltrials.gov NCT01015339: https://clinicaltrials.gov/ct2/show/NCT01015339).

### Patients

Eligible participants were aged ≥ 18 years with histologically confirmed gastric or gastroesophageal junction (GEJ) adenocarcinoma; previously untreated metastatic or unresectable disease; one or more measureable lesions according to the Response Evaluation Criteria in Solid Tumors (RECIST) 1.0 criteria; Karnofsky performance score (KPS) ≥ 70; life expectancy of ≥ 3 months; and acceptable results of the pre-specified hematological and biochemical tests. Previous neo-adjuvant or adjuvant treatments for gastric cancer (except taxanes) were permissible if completed > 6 months, or > 1 year if they comprised capecitabine (not more than 2 cycles) and/or cisplatin (total dose not more than 300 mg/m^2^) before study initiation. No prior radiotherapy was permitted, except for non-target lesions if completed > 4 weeks before study initiation. Key exclusion criteria included brain metastasis, long-term systemic steroid treatment, significant clinical symptoms of cardiac diseases within the last 6 months, known allergy to any study drugs, inability to receive oral medication, pregnancy or lactation period, use of any investigational agent within the past 28 days, any other previous malignancy within 5 years except non-melanoma skin cancer or in situ cervix carcinoma, and issues due to legal incapacity.

### Procedures

Eligible patients were randomly assigned in a 1:1 ratio using a SAS 9.1.3 generated sequence. Stratified randomization by minimization was performed based on KPS (≥ 80/< 80), resection of primary tumor (performed/not performed), weight loss within last 3 months (≥ 5%/< 5%), primary tumor site at the GEJ (yes/no). Nurses dispersed the drugs according to the randomization list.

In PACX group, a total of 4 cycles PACX therapy followed by capecitabine monotherapy for maintenance was administered. For each cycle, paclitaxel was administered intravenously at 80 mg/m^2^ over 3 h on days 1 and 8; capecitabine (Xeloda; F. Hoffmann-La Roche Ltd., Basel, Switzerland) was administered orally at 1000 mg/m^2^ twice daily on days 1–14, followed by a 7-day rest interval (Online Resource 1). Patients received standard anti-hypersensitivity prophylaxis treatment including 10 mg of intravenous dexamethasone, 40 mg of intramuscular diphenhydramine, and 400 mg of intravenous cimetidine 30 min before each paclitaxel administration. After this double-drug regimen, patients received capecitabine monotherapy at the same dosage and schedule as the maintenance therapy until disease progression or development of unendurable adverse events (AEs) or death.

In XP group, a total of 6 cycles XP therapy were planned. Cisplatin was administered intravenously at 80 mg/m^2^ for 2 h on day 1 with hydration and standard delayed emesis prophylaxis treatment per cycle. Capecitabine was administered at the same dosage and schedule as in PACX group (Online Resource 1). In both groups, cycles were repeated every 3 weeks until progression, occurrence of unendurable AEs, consent withdrawal, or a total of 4 or 6 cycles of therapy for PACX and XP, respectively, whichever was earlier.

Dose adjustment was based on the severity of hematologic and non-hematologic toxicity, graded according to the Common Terminology Criteria for Adverse Events version 3.0 (CTCAE v3.0) and the pre-decided dose modification scheme as specified in the study protocol. Simultaneous dose reduction of capecitabine and paclitaxel was avoided unless patients developed severe AEs.

Routine evaluation (physical examination, vital signs, KPS, laboratory hematological and/or serum chemistry, recording and grading of AEs) of patients was conducted on a weekly basis during therapy. Tumor response was evaluated by computerized tomography or magnetic resonance imaging every 6 and 9 weeks during treatment and follow-up, respectively, according to the RECIST 1.0 criteria. QoL was evaluated using the European Organization for Research and Treatment of Cancer QoL Questionnaire (EORTC QLQ)-C30 and EORTC QLQ-STO22 questionnaires at the beginning of each CT cycle until progression, occurrence of unendurable AEs, consent withdrawal, or completion of a total 4 or 6 cycles of therapy for PACX and XP, respectively, whichever was earlier. After disease progression, patients were followed-up every 12 weeks to determine survival until death or the completion of the study.

### Outcomes

The primary endpoint was progression-free survival (PFS), defined as the duration from the date of signing informed consent forms to the first observed disease progression (per imaging examination) or any-cause death, whichever was earlier. The secondary endpoints included disease control rate (DCR) representing the summation of complete response (CR), partial response (PR), and stable disease (SD) rates; objective response rate (ORR), the proportion of total tumor responses (CR and PR) relative to treated patients; overall survival (OS), the duration from the date of signing informed consent forms to death; AEs; serious AEs (SAEs) and QoL.

### Statistical analysis

An overall sample size of 320 subjects (160 in each group) will achieve 80% power at a 0.05 significance level to detect a hazard ratio of 0.69 when the control group median PFS is 4.5 months.

Safety analysis was performed on the safety set (SS) including all patients who had received treatment at least once, and efficacy analysis was performed on intention-to-treat (ITT) set including all patients who intended to receive treatment. An interim safety analysis was scheduled after enrolment of 160 cases. The final analysis of all the endpoints was performed 12 months after the last treatment of the last patient or after approximately 75% of patients had died, whichever occurred first.

PFS and OS were assessed using the Kaplan–Meier method and compared by the log-rank test. ORR and DCR were presented with the corresponding two-sided 95% confidence interval (CI) and analyzed by logistic regression. AEs, SAEs, and drug dose exposure were analyzed using descriptive methods. The *p* values were two-sided and *p* < 0.05 was considered statistically significant. All statistical analyses were conducted using SAS (version 9.1.3).

## Results

### Patient characteristics

**Fig. 1:**
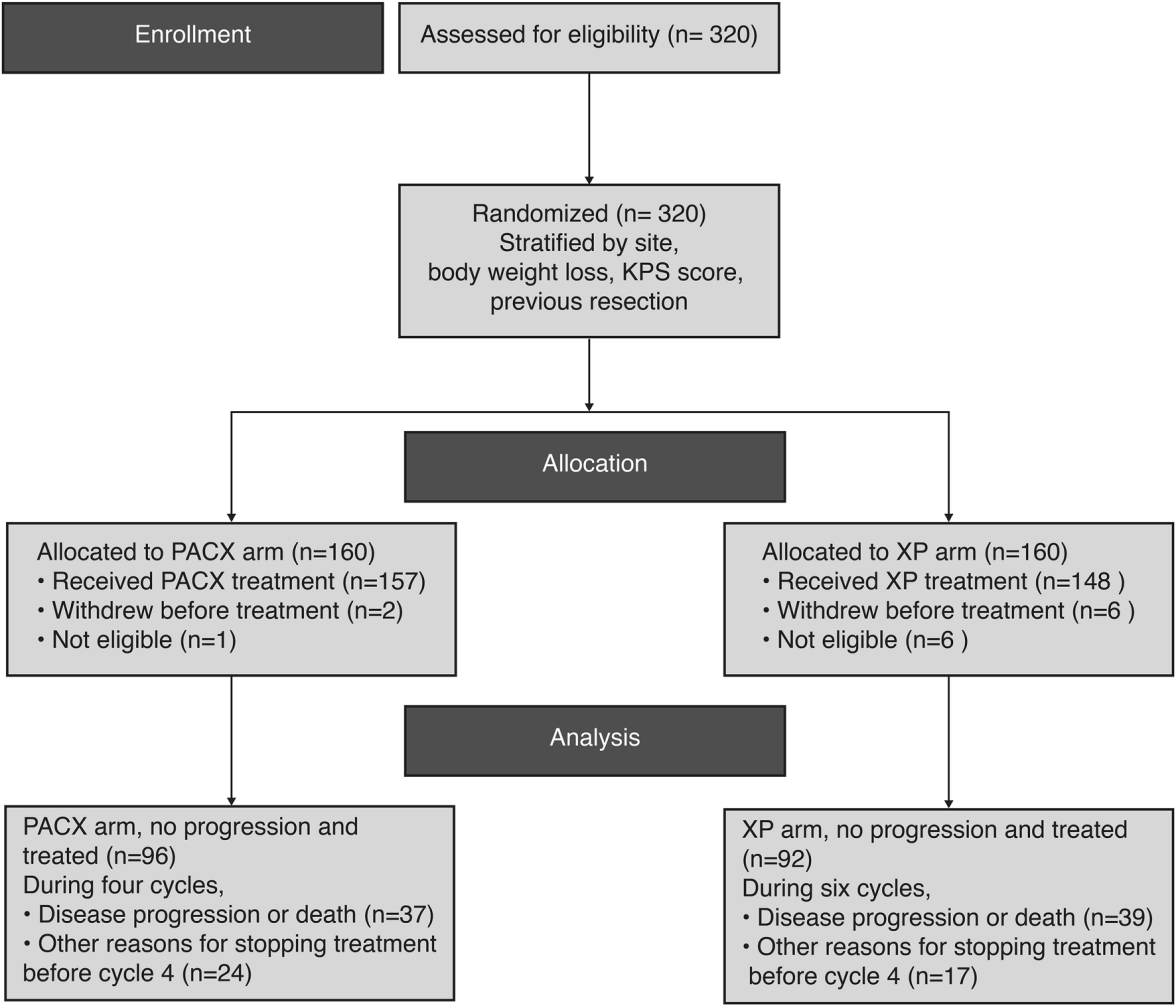
Patient disposition. PACX combination therapy of paclitaxel and capecitabine followed by capecitabine monotherapy as maintenance therapy, XP cisplatin and capecitabine combination therapy

A total of 320 patients were enrolled and randomized in 1:1 ratio to PACX or XP groups (Fig. 1). A total of 252 (78.8%) deaths occurred and 8 patients were lost during follow-up in each arm. The ITT set included 160 patients from each treatment group. The SS set comprised 157 and 148 patients from PACX group and XP group, respectively. The baseline characteristics were well-balanced in both groups (Table 1).

**Table 1:** Baseline characteristics (intention-to-treat set)

	PACX (*n* = 160)	XP (*n* = 160)	*p* value
Age (years), mean	56.6 (11.3)	56.2 (10.9)	0.75
Men	115 (71.9%)	118 (73.8%)	0.71
BSA (m^2^), mean	1.7 (0.2)	1.6 (0.2)	0.60
Weight loss, mean	4.9% (6.4)	5.5% (6.9)	0.42
KPS score, mean	86.4 (7.0)	86.1 (7.4)	0.74
Resection of primary tumor	51 (31.9%)	50 (31.3%)	0.90
Primary tumor at the GEJ	68 (42.5%)	63 (39.4%)	0.57
Metastatic status at diagnosis			
Locally advanced	14 (8.8%)	20 (12.5%)	0.28
Liver metastasis	71 (44.4%)	76 (47.5%)	0.57
Peritoneal metastasis	8 (5.0%)	4 (2.5%)	0.24
Metastasis at other sites	72 (45.0%)	62 (38.8%)	0.26
Number of metastatic sites			
1–2	59 (36.9%)	62 (38.7%)	0.45
> 2	101 (63.1%)	98 (61.3%)	0.34
Lauren classification			
Intestinal type	40 (25.0%)	31 (19.4%)	0.82
Diffused type	40 (25.0%)	35 (21.9%)	
Mixed type	11 (6.9%)	7 (4.4%)	
Missing data	69 (43.1%)	87 (54.4%)	0.04
Second or later line chemotherapy: Yes	56 (35.7%)	47 (31.8%)	0.47

Data are *n* (%) or mean (SD)

*BSA* body surface area, *GEJ* gastroesophageal junction, *KPS* Karnofsky performance score, *PACX* combination therapy of paclitaxel and capecitabine followed by capecitabine monotherapy as maintenance therapy, *SD* standard deviation, *XP* cisplatin and capecitabine combination therapy

The median treatment cycle for the double-drug regimen was 4 and 5 cycles in PACX and XP groups, respectively. The median percentage of actual dose administered relative to the planned dose was > 80% for capecitabine in both groups, 96.8% for paclitaxel in PACX group, and 98% for cisplatin in XP group. In total, 26 (16.6%) and 40 (27.0%) patients had pac2litaxel and cisplatin dose adjustment (*p* = 0.02), 31 (19.7%) and 40 (27.0%) patients had capecitabine dose adjustment (*p* = 0.12), and drug administration was postponed in 101 (64.3%) and 108 (73.0%) patients (*p* = 0.07), in PACX and XP group, respectively. In PACX group, 61 (38.9%) patients did not receive capecitabine monotherapy: *n* = 37, disease progression; *n* = 10, refusal to maintain; *n* = 8, unknown reasons; *n* = 5, poor tolerance; *n* = 1, surgery. The remaining 96 patients in PACX group received capecitabine monotherapy, and the median number of maintenance cycles was 4. The proportion of patients receiving second-line therapy was similar in both groups (PACX group: *n* = 56, 35.7%; XP group: *n* = 47, 31.8%; *p* = 0.465). The second-line treatment regimens in both groups were similar (*p* = 0.423). 42.3% (24/56) patients in PACX group received oxaliplatin-based chemotherapy, while 30.0% (14/47) patients in XP group received docetaxel-based chemotherapy.

### Efficacy

**Fig. 2:**
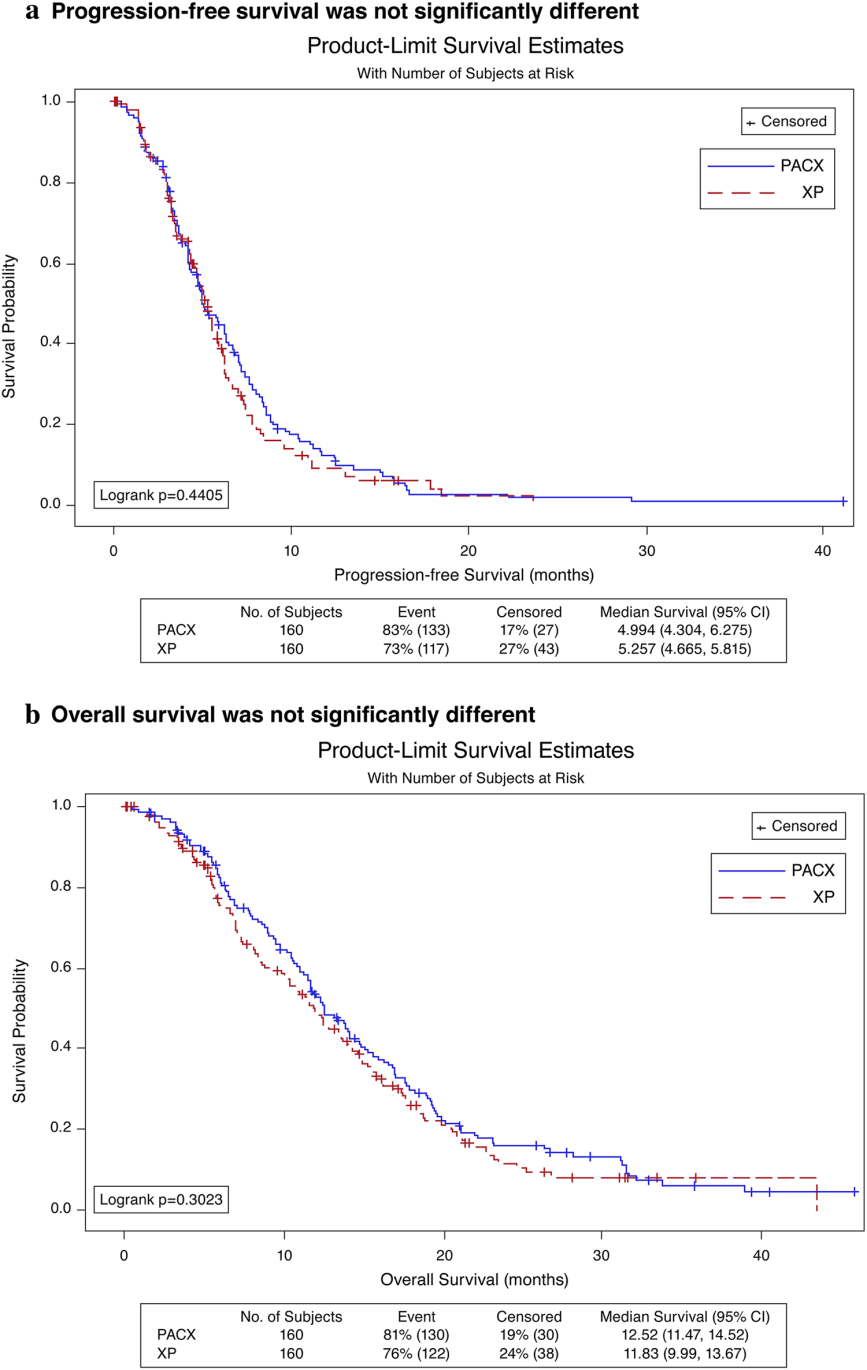
Progression-free survival and overall survival. **a** Progression-free survival was not significantly different; **b** overall survival was not significantly different. *CI* confidence interval, *PACX* combination therapy of paclitaxel and capecitabine followed by capecitabine monotherapy as maintenance therapy, *XP* cisplatin and capecitabine combination therapy

The median follow-up time was 31.4 (95% CI 27.7–35.8) months. Median PFS, the primary endpoint of this study, was 5.0 (95% CI 4.3–6.3) months in PACX group and 5.3 (95% CI 4.7–5.8) months in XP group (hazard ratio 0.906; 95% CI 0.706–1.164; *p* = 0.44, Fig. 2a). Median OS was 12.5 (95% CI 11.5–14.5) months in PACX group and 11.8 (95% CI 10.0–13.7) months in XP group (hazard ratio 0.878; 95% CI 0.685–1.125; *p* = 0.30, Fig. 2b). ORR was significantly higher in PACX group than XP group (43.1% versus 28.8%, *p* = 0.012), whereas DCR (77.5 versus 72.5%, *p* = 0.75) was not significantly different (Table 2). Median PFS, median OS, ORR, and DCR were not significantly different between both groups for subgroup analysis of patients with different Lauren classification and different types of metastases.

**Table 2:** Objective response rate and disease control rate (intention-to-treat set)

	PACX (*n* = 160)	XP (*n* = 160)	*p* value^a^
Response rate			0.022
Complete Response	3 (1.3%)	6 (3.6%)	
Partial response	66 (41.3%)	40 (25.0%)	
Stable	55 (34.4%)	70 (43.8%)	
Progression	16 (10.0%)	14 (8.8%)	
ORR			0.01
*n*	69 (43.1%)	46 (28.8%)	
95% CI	37.3%–53.7%	24.3%–40.0%	
OR (95% CI)	1.9 (1.2–3.3)		
DCR			0.75
*n*	124 (77.5%)	116 (72.5%)	
95% CI	74.5%–87.4%	72.6%–86.2%	
OR (95% CI)	1.1 (0.6–2.1)		

Data are *n* (%) unless otherwise indicated

*CI* confidence interval, *DCR* disease control rate, *OR* odds ratio, *ORR* objective response rate, *PACX* combination therapy of paclitaxel and capecitabine followed by capecitabine monotherapy as maintenance therapy, *XP* cisplatin and capecitabine combination therapy

^a^Response rate, ORR, and DCR of PACX and XP arms were compared by logistic regression analyses using the four stratification factors as independent variables

### Safety and quality of life

**Fig. 3:**
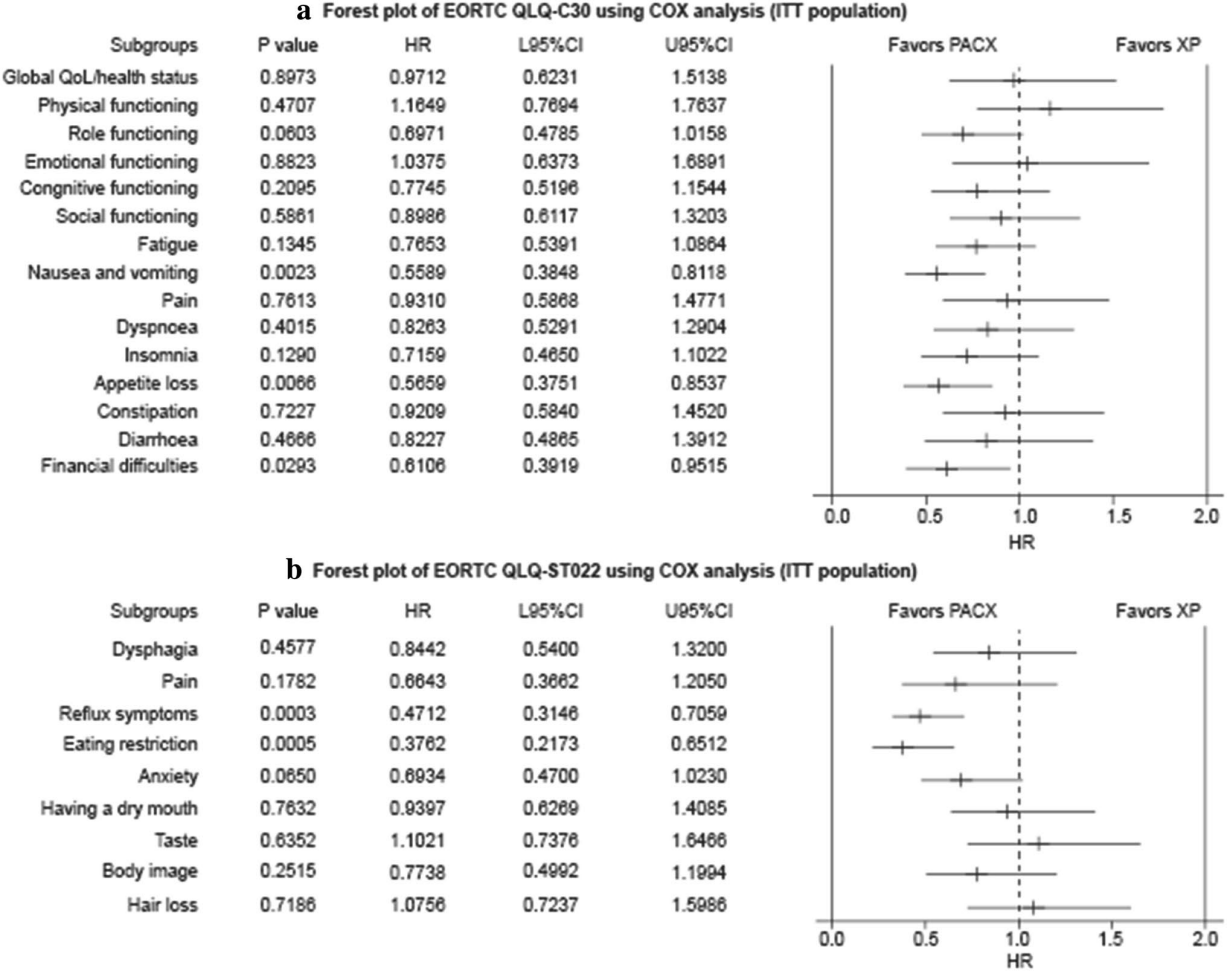
Quality of life: time to first deterioration (ITT population). **a** Forest plot of EORTC QLQ-C30 using COX analysis (ITT population); **b** forest plot of EORTC QLQ-ST022 using COX analysis (ITT population). *CI* confidence interval, *EORTC QLQ* European Organization for Research and Treatment of Cancer Quality of Life Questionnaire, *HR* hazard ratio, *ITT* intention-to-treat, *PACX* combination therapy of paclitaxel and capecitabine followed by capecitabine monotherapy as maintenance therapy, *XP* cisplatin and capecitabine combination therapy

Forest plot of time to first deterioration assessed by EORTC QLQ-C30 for PACX group was significantly shorter than that of XP group, suggesting significantly improved QoL in PACX group than in XP group (Fig. 3a). The EORTC QlQ-STO22 was also significantly different as to some symptoms between both groups (Fig. 3b).

During the study, 1 patient in XP group erroneously received the treatment regimen of PACX group. Thus, safety was evaluated in 158 patients of PACX group and 147 patients of XP group. Blood and lymphatic system disorders and gastrointestinal system disorders were the most common treatment-related AEs in both groups. The incidences of treatment-related leukopenia, thrombocytopenia, nausea, vomiting, lack of appetite, and vascular disorders were significantly higher in XP group than PACX group (all *p* < 0.05, Table 3). Alopecia and musculoskeletal and connective tissue disorders were more frequent in PACX group than in XP group (all *p* < 0.05, Table 3). Analysis of treatment-related AEs at level III or IV revealed that the incidences of anemia, thrombocytopenia, vomiting and nausea were significantly higher in XP group than PACX group (all *p* < 0.05, Table 3). 6 (3.8%) patients in PACX group and 4 (2.7%) patients in XP group developed treatment-related SAEs (Table 3).

**Table 3:** Treatment-related adverse events, common terminology criteria for adverse events version 3.0 grade ≥ 3 treatment-related adverse events, and treatment-related serious adverse events

	Any treatment-related AEs*			Grade ≥ 3 treatment-related AEs			Any treatment-related SAEs			
	PACX *n* = 158	XP *n* = 147	*p* value	PACX *n* = 158, (%)	XP *n* = 147, (%)	*p* value		PACX *n* = 158, (%)	XP *n* = 147, (%)	*p* value
Patients reporting AEs	132 (83.5%)	130 (88.4%)	0.22	54 (34.2)	59 (40.1)	0.28	Patients reporting SAEs	6 (3.8)	4 (2.7)	0.60
Blood and lymphatic system disorders	104 (65.8%)	108 (73.5%)	0.15	43 (27.2)	37 (25.2)	0.69	Drug hypersensitivity	3 (1.9)	0 (0.0)	0.10
Leukopenia	77 (48.7%)	99 (67.3%)	0.001	20 (12.7)	14 (9.5)	0.38	Agranulocytosis	1 (0.6)	0 (0.0)	0.33
Neutropenia	78 (49.4%)	79 (53.7%)	0.45	33 (20.9)	23 (15.6)	0.24	Neurotoxicity	1 (0.6)	0 (0.0)	0.33
Anemia	40 (25.3%)	45 (30.6%)	0.30	3 (1.9)	10 (6.8)	0.03	Multiple organ failure	1 (0.6)	0 (0.0)	0.33
Thrombocytopenia	6 (3.8%)	26 (17.7%)	< 0.0001	1 (0.6)	7 (4.8)	0.02	Diarrhea	1 (0.6)	0 (0.0)	0.33
Gastrointestinal system disorders	60 (38.0%)	92 (62.6%)	< 0.0001	8 (5.1)	18 (12.2)	0.03	Thrombocytopenia	0 (0.0)	1 (0.7)	0.30
Nausea	26 (16.5%)	61 (41.5%)	< 0.0001	3 (1.9)	12 (8.2)	0.01	Hepatic function abnormal	0 (0.0)	1 (0.7)	0.30
Vomiting	23 (14.6%)	64 (43.5%)	< 0.0001	4 (2.5)	14 (9.5)	0.01	Angioedema	0 (0.0)	1 (0.7)	0.30
Hypophagia	15 (9.5%)	32 (21.8%)	0.003	0 (0.0)	0 (0.0)	NA	Sudden death	0 (0.0)	1 (0.7)	0.30
Diarrhea	7 (4.4%)	9 (6.1%)	0.51	0 (0.0)	0 (0.0)	NA	Hemorrhage	0 (0.0)	1 (0.7)	0.30
Vascular disorders	1 (0.6%)	6 (4.1%)	0.05	0 (0.0)	0 (0.0)	NA	–	–	–	–
Alopecia	14 (8.9%)	1 (0.7%)	0.001	0 (0.0)	0 (0.0)	NA	–	–	–	–
Musculoskeletal and connective tissue disorders	6 (3.8%)	0 (0.0%)	0.02	0 (0.0)	0 (0.0)	NA	–	–	–	–
Abnormal laboratory test	31 (19.6)	32 (21.8)	0.25	1 (0.6)	5 (3.4)	0.08	–	–	–	–
Blood bilirubin elevation	–	–	–	0 (0.0)	3 (2.0)	0.07	–	–	–	–
Skin and subcutaneous tissue disorders	–	–	–	3 (1.9)	3 (2.0)	0.93	–	–	–	–
Palmar-plantar erythrodysesthesia syndrome	–	–	–	2 (1.3)	2 (1.4)	0.94	–	–	–	–
General disorders and administration site conditions	–	–	–	2 (1.3)	4 (2.7)	0.36	–	–	–	–
Fatigue	–	–	–	1 (0.6)	4 (2.7)	0.15	–	–	–	–

All data are *n* (%)

*AE* adverse event, *NA* not applicable, *PACX* combination therapy of paclitaxel and capecitabine followed by capecitabine monotherapy as maintenance therapy, *SAE* serious adverse event, *XP* cisplatin and capecitabine combination therapy

*Only the AEs with prevalence ≥ 3% in either group are included in ‘Any treatment related AEs’

## Discussion

In this randomized phase III study, PACX regimen did not show longer PFS, OS, and DCR, but significantly higher ORR and QoL compared with XP regimen. In spite of the negative result of efficacy, the PACX regimen was related with significantly lower incidences of hematologic toxicity such as leukopenia and thrombocytopenia and gastrointestinal AEs such as nausea, vomiting and lack of appetite, than the XP regimen.

Although PFS is not always strictly related with OS in first-line therapy of AGC, it is often used as an alternative, especially when progression is likely to be related to symptomatology. Also, considering convenience and economical factors, we selected PFS as the primary endpoint. Capecitabine monotherapy was introduced in PACX regimen after disease control; we thus supposed PFS might be prolonged by continual exposing of chemotherapeutical agent compared with fixed cycles of XP. This study was planned as a superior design, but not as a non-inferior design.

The efficacy and safety of combination of paclitaxel and capecitabine as a first-line treatment for AGC have been investigated in previous phase II trials. The results of the current study, which showed a median PFS of 5.0 months and a median OS of 12.5 months in PACX group, were similar to previous phase II clinical trial findings with capecitabine maintenance as well [13, 14], but longer than that of Yuan’s retrospective data [15]. Since the number of patients having second-line and follow-up treatments are not statistically different between two groups, the longer median PFS and OS in this study compared with Yuan’s data might be related with the addition of capecitabine monotherapy following the double-drug combination therapy, indicating that single-drug monotherapy following a first-line CT for AGC might improve patient survival.

Preclinical studies have demonstrated that combination of capecitabine and paclitaxel had synergistic antitumor activity [16, 17]. Capecitabine is finally converted to fluorouracil by thymidine phosphorylase, which is expressed at higher levels in tumor tissue, leading to the accumulation of fluorouracil in tumor tissue [18, 19]. Sequential exposure to paclitaxel following 5-FU exerted additive cytotoxic effects in human carcinoma cell lines by up-regulating thymidine phosphorylase activity [16, 17]. For the first time, in this current randomized control trial, we found that combination therapy of paclitaxel and capecitabine followed by capecitabine monotherapy resulted in significantly higher ORR than combination therapy of cisplatin and capecitabine for AGC. Despite higher ORR, PACX regimen showed similar PFS, OS, and DCR with XP regimen in advanced gastric cancer. This might primarily be due to its genetic complexity and heterogeneity. Therefore, the identification of novel and specific markers to predict PACX treatment in gastric cancer is important.

The safety profile evaluated in previous studies consistently shows that combination of paclitaxel and capecitabine as a first-line therapy for AGC was related with mild AEs, and the most common grade 3–4 AEs included neutropenia, leukopenia, and vomiting [13–15]. In this study, comparison of the safety profile revealed that the PACX regimen was associated with significantly reduced incidences of blood and lymphatic system disorders and gastrointestinal system disorders compared with the XP regimen. Consistently, QoL was also significantly improved in patients receiving PACX regimen than those receiving XP regimen.

It can be argued that 4 cycles of combination therapy in PACX are not enough, thus limiting the efficacy and explaining the better tolerance. It is not known whether more cycles of paclitaxel combined with capecitabine before monotherapy maintenance could further improve survival. In future clinical practice and clinical study, it is at least reasonable to continue combination therapy until accumulated peripheral neuropathy.

To our knowledge, this study is the first multicenter phase III randomized control trial to compare the efficacy and safety of PACX regimen. To maximally balance patient clinical characteristics between both groups at randomization, we used four stratification factors (KPS, previous resection of primary tumor, weight loss within 3 months of enrolment, and primary tumor site). The well-balanced baseline data of this study suggested that our randomization strategy was effective. However, there are limitations in this study. The number of patients (96/160, 60%) in PACX group who received capecitabine maintenance therapy was relatively small. Moreover, expression of HER2 has not been tested in patients because this study was initiated before the publication of ToGA trial. Results of the current study may only be restricted to AGC patients without over-expressed HER2. Therefore, the results of our subgroup analyses need to be validated in larger clinical trials with the consideration of HER2 expression. Further studies are needed to evaluate the extrapolation of results from this trial to real-world settings.

In conclusion, the PACX regimen did not improve the PFS and OS compared with the XP regimen in patients with AGC. But, patients receiving PACX showed significantly higher ORR, improved QoL, and reduced incidences of AEs such as blood and lymphatic system disorder and gastrointestinal disorders than those receiving XP. Thus, PACX is an effective treatment with milder toxicities, but so far it may not be able to replace the first-line standard chemotherapy of XP. Future research of biomarkers for improving efficacy in identifying highly responsive patients is warranted.

## Electronic supplementary material

Below is the link to the electronic supplementary material.

Supplementary material 1 (PDF 144 kb)
