# Supplementary material for: A multicenter, randomized trial comparing efficacy and safety of paclitaxel/capecitabine and cisplatin/capecitabine in advanced gastric cancer
Source: Gastric Cancer. 2018 Feb 27;21(5):782–91. doi: 10.1007/s10120-018-0809-y (PMC6097104; doi:10.1007/s10120-018-0809-y)

Article title: A multicenter, randomized, trial comparing efficacy and safety of paclitaxel/capecitabine and cisplatin/capecitabine in advanced gastric cancer

Journal name:

Author Names: Zhihao Lu[1†], MD; Xiaotian Zhang[1†], MD; Wei Liu[2†], MD; Tianshu Liu[3†], MD; Bing Hu[4], MD; Wei Li[5], MD; Qingxia Fan[6], MD; Jianming Xu[7], MD; Nong Xu[8], MD; Yuxian Bai[9], MD; Yueyin Pan[10], MD; Qing Xu[11], MD; Wei Bai[12], MD; Li Xia[13], MD; Yong Gao[14], MD; Wenling Wang[15], MD; Yongqian Shu[16], MD; Lin Shen[1*], MD

[†]first four authors have contributed equally

Corresponding author:

Prof. Lin Shen

Key laboratory of Carcinogenesis and Translational Research (Ministry of Education/Beijing), Department of Gastrointestinal Oncology,

Peking University Cancer Hospital and Institute

#52 Fucheng Road, Haidian District, Beijing, 100142, P. R. China

E-mail: linshenpku@163.com

Online Resource 1. Study design and dosing/schedule details.

a. Study design;

b. Dosing and schedule of single cycle of PACX regimen

c. Dosing and schedule of single cycle of XP regimen

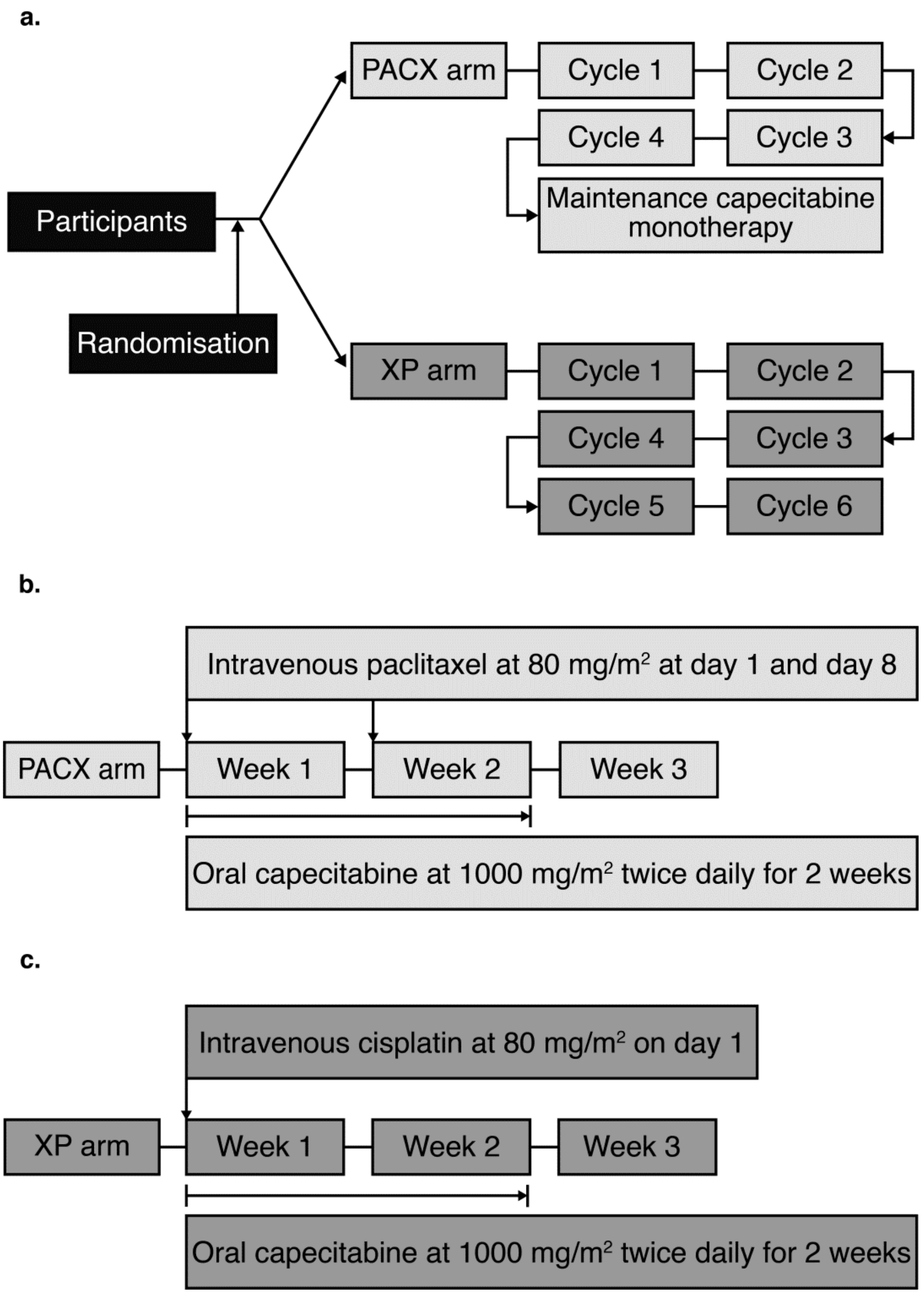

Supplement: Supplementary file 1 — Supplementary material 1 (PDF 144 kb) [file 10120_2018_809_MOESM1_ESM.pdf]
